# Propentofylline and Interleukin-4 Modulate Lesion-Associated Myeloid Responses and Improve Functional Recovery After Spinal Cord Injury

**DOI:** 10.3390/cells15070625

**Published:** 2026-03-31

**Authors:** Mousumi Ghosh, Amir-Hossein Bayat, Keeley S. Garvey, Tolani Oshinusi, Thomas De Leon, Jacqueline Sagen, Damien D. Pearse

**Affiliations:** 1 The Miami Project to Cure Paralysis, The University of Miami Miller School of Medicine, Miami, FL 33136, USA; axm8710@med.miami.edu (A.-H.B.); kgarvey@students.ocom.org (K.S.G.); tolanioshinusi@gmail.com (T.O.); thomastheleon@gmail.com (T.D.L.); jsagen@med.miami.edu (J.S.); dpearse@med.miami.edu (D.D.P.); 2 The Department of Neurological Surgery, The University of Miami Miller School of Medicine, Miami, FL 33136, USA; 3 The Neuroscience Program, The University of Miami Miller School of Medicine, Miami, FL 33136, USA; 4 The Interdisciplinary Stem Cell Institute, The University of Miami Miller School of Medicine, Miami, FL 33136, USA

**Keywords:** spinal cord injury, microglia, myeloid cells, neuroinflammation, propentofylline, interleukin-4, immunomodulation, tissue preservation

## Abstract

**Highlights:**

**What are the main findings?**

- Systemic co-treatment with propentofylline (PPF) + interleukin-4 (IL-4), started ≤1 h post-contusion; daily × 14 days, produced a greater improvement in gross (BBB) and skilled locomotion than vehicle, or either monotherapy, after thoracic SCI in adult rats.
- The combination most strongly remodeled lesion-associated myeloid signaling, suppressing chronic p-p38 MAPK while sustaining the expression of reparative markers (ARG1, CD206), and was associated with reduced cavitation and trends toward greater gray and white matter preservation.

**What are the implications of the main findings?**

- Pairing an inflammation-dampening glial modulator, PPF, with a reparative polarizing cue, IL-4, was associated with reduced inflammatory signaling, increased repair-associated marker expression, and improved locomotor outcomes after SCI.
- Because both agents were delivered systemically using an early short-duration dosing window, the present findings justify additional preclinical investigation of this combinatorial approach, with particular emphasis on comprehensive immune profiling, safety, pharmacokinetics, and translational validation.

**Abstract:**

Spinal cord injury (SCI) triggers a secondary injury cascade characterized by persistent innate immune activation, chronic neuroinflammation, and progressive tissue loss that limits functional recovery. Here, we evaluated a systemic combination treatment using propentofylline (PPF), a glial modulator, together with interleukin-4 (IL-4), a cytokine associated with repair-related myeloid responses. In vitro, PPF enhanced IL-4-dependent induction of arginase-1 (ARG1) in TNFα-primed BV2 microglia. In vivo, adult Fischer rats of both sexes received vehicle, PPF, IL-4, or combined PPF + IL-4 beginning within 1 h after moderate T8 contusive SCI and continuing daily for 14 days. Locomotor recovery was assessed longitudinally for 8 weeks, followed by histological and immunohistochemical analyses. Combined PPF + IL-4 treatment produced the greatest improvement in gross and skilled locomotor recovery compared with vehicle, or either monotherapy. At 8 weeks post-SCI, the combined therapy aligned with a reduction in chronic lesion-associated p-p38 MAPK, decreased pP65 NFkB (RelA) activation, increased expression of reparative factors ARG1 and CD206, as well as reduced lesion cavitation and trends toward greater gray and white matter preservation. Stratification of functional data by sex showed BBB improvements with combined PPF + IL-4 in both males and females after SCI. Together, these findings show that combined systemic PPF and IL-4 treatment was associated with improved functional recovery, reduced lesion cavitation, and changes in lesion-associated molecular and histological endpoints after SCI, supporting further preclinical investigation.

## 1. Introduction

Traumatic spinal cord injury (SCI) is a devastating neurological condition that results in permanent motor, sensory, and autonomic dysfunction, with profound consequences for quality of life and long-term independence [1,2]. Despite advances in surgical decompression, stabilization, and rehabilitation, there are currently no pharmacological therapies that reliably restore lost neurological function after SCI [3,4]. Functional outcome is determined not only by the severity of the initial mechanical insult but also by a prolonged and evolving secondary injury cascade that unfolds over days to weeks following trauma. This secondary injury phase is characterized by neuroinflammation, oxidative stress, excitotoxicity, vascular dysfunction, and progressive loss of neurons, oligodendrocytes, and axons, ultimately leading to lesion expansion and chronic neurological deficits [2,5,6]. Among these processes, neuroinflammation is a central and sustained driver of secondary tissue damage after SCI [7,8]. Resident microglia and infiltrating monocyte-derived macrophages are rapidly activated following injury and adopt predominantly pro-inflammatory phenotypes that amplify tissue damage through the release of cytokines, chemokines, reactive oxygen species, and proteases [9,10,11,12]. Persistent phenotypic change of these innate immune cells towards a proinflammatory state is strongly associated with axonal degeneration, demyelination, lesion cavitation, and impaired functional recovery.

Importantly, microglia and macrophages are not intrinsically detrimental and retain the capacity to adopt reparative phenotypes that support tissue preservation, debris clearance, angiogenesis, and remyelination [13]. These reparative states are often referred to as alternatively activated or pro-repair phenotypes characterized by expression of markers such as the mannose receptor (CD206) and the secretion of anti-inflammatory and trophic factors [13,14,15]. However, following SCI, reparative polarization is often insufficient, delayed and/or transient, allowing chronic proinflammatory signaling to dominate the injury milieu [16,17,18]. Thus, therapeutic strategies that both suppress detrimental inflammatory signaling and actively promote sustained reparative immune polarization represent a promising approach for limiting secondary injury and enhancing recovery [13,19,20].

Interleukin-4 (IL-4) is a potent immunoregulatory cytokine that promotes alternative activation of microglia and macrophages and has been shown to enhance tissue repair after central nervous system (CNS) injury [21,22,23,24]. Nevertheless, IL-4 administration alone has demonstrated limited efficacy in the chronic phase of SCI, likely due to persistent inflammatory signaling that constrains stable reparative polarization [13,22,25]. Propentofylline (PPF), a xanthine derivative with well-documented neuroprotective and immunomodulatory properties, suppresses glial activation and attenuates inflammatory signaling pathways, including p-p38 MAPK, in both in vitro and in vivo models [26,27,28]. While PPF effectively dampens inflammatory activation, it does not independently drive robust reparative immune phenotypes [29].

In the present study, we examined whether acute systemic co-administration of PPF and IL-4, initiated within one hour of injury and continued during the early post-SCI period, could provide additive or complementary effects on gross and fine locomotor recovery, chronic inflammatory signaling, reparative myeloid polarization, and tissue preservation. We hypothesized that PPF would primarily dampen inflammatory pathway activity, whereas IL-4 would provide reparative instruction to lesion-associated myeloid cells. This work therefore tested whether combined treatment could improve outcomes through complementary pathway engagement rather than through a directly demonstrated receptor-level synergistic interaction. This work introduces an innovative combinatorial immunomodulatory strategy that concurrently targets inflammatory signaling and immune phenotype, offering a mechanistically supported and translationally feasible approach for improving outcomes after SCI.

## 2. Materials and Methods

### 2.1. Reagents

Rat IL-4 (Recombinant Protein, Cat #400-04-20UG, PeproTech, Cranbury, NJ, USA) and rat TNF-α (Recombinant Protein, Cat # 315-01A-20UG, PeproTech, Cranbury, NJ, USA) were purchased from PeproTech (Rocky Hills, NJ, USA). PPF was obtained from Cayman Chemicals (Item No. 29431, Ann Arbor, MI, USA).

The different primary antibodies; their hosts, sources, and dilutions employed for immunohistochemistry; and Immunoblot analysis are provided in Table 1 below.

### 2.2. BV2 Microglial Cell Culture

In vitro experiments were conducted using BV2 microglia, an immortalized microglial cell line established from the C57BL/6 mouse strain [30], which maintains many of the functional properties of primary microglia [31]. BV2 microglia were maintained under standard incubation conditions (37 °C, 5% CO_2_) in Dulbecco’s Modified Eagle Medium (DMEM; Gibco, Life Technologies Corporation, Grand Island, NY, USA) containing 10% heat-inactivated fetal bovine serum (FBS; HyClone, Logan, UT, USA) and penicillin–streptomycin (100 U/mL; Sigma-Aldrich, Saint Louis, MO, USA), following previously described procedures [13,32]. For downstream biochemical analyses, BV2 microglial cells were plated in six-well plates at a density of 5  ×  10^4^ cells/well and allowed to reach near-confluent levels (50–60%). Cells were subsequently treated with cytokines and/or PPF, alone or in combination, as required.

### 2.3. Cyclic AMP Assay

Changes in intracellular cyclic AMP concentration were assessed following exposure of BV2 microglial cultures to increasing concentrations of PPF (0–1000 μM) for 24 h as conducted previously [33,34]. These concentrations were selected to interrogate signaling mechanisms rather than to model in vivo pharmacokinetics. Cells were harvested by lysis in 1X cell lysis buffer (Cell Signaling Technology Inc., Danvers, MA, USA) supplemented with the phosphodiesterase inhibitors IBMX (0.5 mM; Tocris, Ellisville, MI, USA) and rolipram (100 μM; Sigma, Saint Louis, MO, USA), followed by incubation on ice for 30 min. Lysates were cleared by centrifugation at 1000× *g* for 10 min at 4 °C. Protein content was quantified using a modified Bradford assay with bovine serum albumin as the reference standard (Bio-Rad Laboratories, Hercules, CA, USA). Clarified supernatants were then assayed for total cyclic AMP using the cyclic AMP-Glo™ detection system (Promega Corporation, Madison, WI, USA) according to the manufacturer’s protocol. Cyclic AMP values were normalized and reported as a percentage of untreated control levels.

### 2.4. Western Blotting

The abundance of specific proteins in primed BV2 cultures were assessed by immunoblot analysis using whole-cell lysates, following previously established protocols in our laboratory [13,33]. Proteins were transferred to nitrocellulose membranes (Bio-Rad Laboratories, Hercules, CA, USA) and incubated with the following primary antibodies: arginase-1 (ARG1; 1:1000; GeneTex, Irvine, CA, USA), and β-actin (1:10,000; Sigma-Aldrich, Saint Louis, MO, USA). Immunoreactive band intensities were quantified by densitometry analysis performed in ImageJ (v1.54g) with background subtraction applied uniformly across membranes. Data shown represent the mean values obtained from three independent experiments.

### 2.5. Animals and In Vivo Experimental Design

Animals. Age-matched adult Fischer rats of both sexes (16–17 weeks old; 180–200 g; Charles River Laboratories, Wilmington, MA, USA) were used in this study. Animals were randomized into four treatment groups (*n* = 25/group; total *N* = 100, including attrition), with a similar male: female ratio. Animals were housed and cared for in accordance with National Institutes of Health standards and the Guide for the Care and Use of Laboratory Animals. All experimental procedures were reviewed and approved by the Institutional Animal Care and Use Committee (IACUC) at the University of Miami (IACUC approvals # 17-023, and 22-126).

**Scheme 1:**
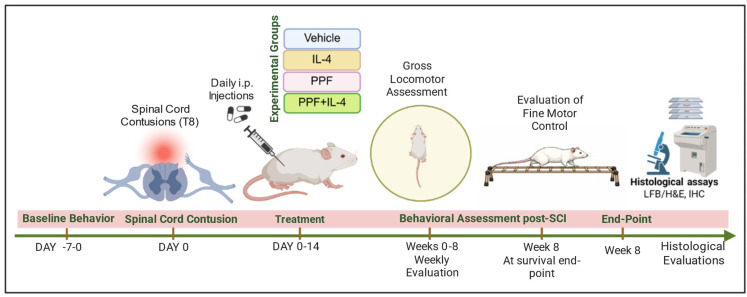
Representation of the experimental timeline illustrating thoracic spinal cord contusion injury, drug administration, behavioral assessments and tissue collection. Animals received intraperitoneal injections of vehicle, PPF, IL-4 or combined treatment, beginning within one hour after induction of SCI and continuing once daily for 14 days. Assessment of gross locomotor function was conducted longitudinally through 8 weeks post-injury followed by tissue processing histology and immunohistochemical analyses at the chronic endpoint of 8 weeks post-SCI/treatment.

Experimental timeline. Thoracic spinal cord contusion injury was induced at T8, followed by daily intraperitoneal administration of vehicle, PPF, IL-4, or PPF+IL-4 beginning within 1 hour post-injury and continuing for 14 days. Gross locomotor recovery was assessed weekly for 8 weeks, fine motor control was evaluated at the 8-week endpoint, and tissues were then collected for histological and immunohistochemical analyses (Scheme 1).

### 2.6. Rat SCI Surgery

Rats were subjected to a moderate thoracic level spinal cord contusion using a calibrated impactor as described below.

Pre-Operative Preparation. Prior to surgery, animals were weighed and anesthetized using 2% isoflurane delivered in 30% oxygen. Depth of anesthesia was verified by assessing respiratory patterns and the absence of corneal and pedal withdrawal reflexes. The dorsal surgical area was shaved and disinfected with chlorhexidine (Phoenix Pharmaceutical Inc., St. Joseph, MO, USA). Artificial tear ointment (Rugby, Livonia, MI, USA) was applied to protect the eyes throughout the procedure [35]. Throughout surgery, animals were maintained on a homeothermic heating system (Harvard Apparatus Ltd., Kent, UK) with rectal temperature feedback to sustain core body temperature at 37 ± 0.5 °C. Spinal cord contusion was performed in female rats when confirmed to be outside the 4–5-day estrous window to minimize the influence of hormone-related variability on post-injury neuroinflammatory responses [35]. Estrous status was assessed pre-operatively using a vaginal impedance meter (Model MK 11, Muromachi Kikai, Tokyo, Japan) as previously described [35]. The probe was lubricated with sterile, non-spermicidal jelly (First Priority, Elgin, IL, USA) prior to insertion. This procedure was used to improve reproducibility for inflammation-related endpoints. Both male and female animals were included in the study. Although the experimental design was not specifically powered to detect sex-by-treatment interactions across all endpoints, behavioral outcomes were additionally analyzed in a sex-stratified manner to assess potential sex-dependent differences in treatment response. For histological and immunohistochemical endpoints, analyses were conducted using pooled male and female cohorts because subgroup sizes within the predefined histological subset were insufficient to support statistically robust sex-disaggregated comparisons.

Experimental Induction of Moderate Thoracic SCI. SCI was produced using the Multicenter Animal Spinal Cord Injury Study (MASCIS) impactor, following established procedures [35,36]. Briefly, a laminectomy at the T8 vertebral level was performed to expose the dorsal thoracic spinal cord while leaving the dura intact. A moderate contusion was then generated by dropping a 10.0 g impactor from a height of 25.0 mm. Injury metrics (impact height, velocity, and compression) were recorded, and animals were excluded if parameters deviated by more than 7% from target values or if compression fell outside the 1.75–2.25 mm range. After injury, the muscle and skin were sutured in layers. Animals recovered in a warm setting and were subsequently pair-housed with free access to food and water. Post-operative management included gentamicin (5 mg/kg, intramuscular, for 7 days), buprenorphine (0.03 mg/kg, subcutaneous, for 3 days), and lactated Ringer’s solution (5 cc, subcutaneous, twice daily as needed) [37]. Bladders were expressed manually twice daily until normal spontaneous urination returned.

### 2.7. Drug Administration and Dosing

PPF was administered intraperitoneally at 10 mg/kg body weight and IL-4 at 30 µg/kg, respectively. Doses were selected based on prior reports demonstrating efficacy in experimental CNS injury paradigms [13,38,39], while minimizing adverse systemic effects. Treatments began within one hour after injury and continued once daily for 14 days. Vehicle-treated animals received daily sterile saline via i.p. injections for the same period. We selected a 14-day dosing paradigm to target the acute-to-subacute post-injury interval, when the secondary injury cascade and lesion-associated myeloid activation are most dynamic and therefore most amenable to therapeutic modulation. Behavioral and histological outcomes were then assessed at 8 weeks post-injury to determine whether this early, time-limited intervention produced sustained chronic benefits.

### 2.8. Functional Assessment

Baseline performance on all behavioral assays was assessed in each animal prior to injury. Following injury, functional outcomes were evaluated at defined time points as described below in schematic 1. Behavioral assessments were conducted by two independent observers who were blinded to the treatment conditions for each subject. Animals were excluded from behavioral analysis if the intended SCI was not achieved due to procedural errors such as off-target impact parameters.

BBB open-field locomotor score. Gross locomotor recovery was assessed weekly using the Basso Beattie and Bresnahan (BBB) locomotor rating scale [40] from 24 h to 8 weeks post-injury as conducted previously in our other studies [37].

Grid-walk test. Descending motor function was evaluated to assess fine motor coordination at baseline (pre-injury) and at study endpoint of 8 weeks post-injury following SCI, using the grid walk-task where the animals’ ability to traverse a 1 m runway consisting of round metal bars separated by irregular gaps (0.5–5 cm) was analyzed, as previously described [37,41]. Skilled walking performance was quantified by analyzing traversal accuracy of hind paw placement and missteps across the runway.

### 2.9. Tissue Processing and Histology

At the survival endpoint of 8 weeks, following SCI, animals were euthanized and perfused for tissue collection. Rats were sacrificed by carbon dioxide inhalation and subjected to transcardial perfusion with 500 mL of cold (4 °C) physiological saline, followed by 400 mL of cold 4% paraformaldehyde in phosphate buffer (0.1 M, pH 7.4). The entire CNS tissues were harvested and post-fixed overnight in 4% paraformaldehyde, then cryoprotected in 30% sucrose using established protocols [35,37]. A 2 cm thoracic spinal cord segment encompassing the lesion epicenter was embedded in M-1 embedding medium (ThermoFisher Scientific, Kalamazoo, MI, USA) and cryosectioned coronally at 20 μm thickness into 20 serial sets using a Leica CM3050S cryostat (Leica Microsystems Inc., Buffalo Grove, IL, USA). Tissue sections were stored at −20 °C until analysis. Histological endpoints were assessed in a predefined, randomly selected subset of animals from each cohort, with selection performed blinded to treatment; sample sizes varied by analysis and ranged from 4 to 6 animals per group, as specified in the corresponding figure legends. For each animal, morphometric analyses were performed on serial transverse sections spanning the lesion region; the exact number/spacing of sections analyzed per animal for each endpoint is specified in the corresponding figure legends. Sections were then stained with hematoxylin (H&E; Modified Harris’s Hematoxylin, Ref. 72711, Eperedia, Kalamazoo, MI, USA) and eosin (Eosin Y 1%; Cat. No. 10143-132, VWR, Radnor, PA, USA), as well as Luxol Fast Blue (LFB; Cat. No. L0294, Sigma-Aldrich, St. Louis, MO, USA), to evaluate tissue morphology, myelin preservation, and lesion cavitation, as previously described [41,42,43]. Slides were cover slipped using CV Mount mounting medium (Leica, Ref. 1404643001, London, UK).

### 2.10. Immunohistochemistry

Immunohistochemistry (IHC) and subsequent quantitative analysis of immune cell-associated markers were carried out using established methods [35,41]. Cryosectioned tissue sections were first subjected to heat-mediated antigen retrieval with IHC-Tek epitope retrieval solution (IHC World, Ellicott City, MD, USA). Sections were then blocked for 1 h at room temperature in PBS containing 2% bovine serum albumin and 0.5% Triton X-100. Primary antibodies specific to the protein targets of interest were applied and incubated overnight at room temperature. After three washes in PBS with 0.1% Tween-20, sections were incubated for 2 h with appropriate Alexa fluorophore-conjugated corresponding secondary antibodies (ThermoFisher Scientific, Waltham, MA, USA). Following additional washes, slides were mounted with micro cover glass (VWR, Radnor, PA, USA) using Prolong Diamond Antifade mounting medium (Invitrogen, Carlsbad, CA, USA) and stored at 4 °C until image acquisition was conducted.

### 2.11. Image Analysis

H&E and LFB-stained sections were imaged on an Olympus BX51 microscope (Olympus Corporation, Tokyo, Japan) using Neurolucida Explorer (MicroBrightField Bioscience, Vermont, USA). For volumetric analysis, serial images were generated as per MBF guidelines and, for each section, the perimeter of the total spinal cord and the normal-appearing gray and white matter were traced on live images in Neurolucida Explorer (2019.2.1). Total cord and normal-appearing gray and white matter volumes were calculated, and lesion/cavity volume was derived as total spinal cord volume minus the summed volumes of normal-appearing gray and white matter; three-dimensional reconstructions were generated from the serial section dataset as previously described [42,43]. Fluorescent images of IHC-labeled tissue sections were obtained using a laser-scanning confocal microscope (Fluoview FV1000; Olympus, Center Valley, PA, USA) or the Dragonfly spinning disk confocal microscope (Andor Technology, Oxford Instruments, Concord, MA, USA). For each immunolabeled marker, images encompassing the injury epicenter were collected from the corresponding stained series. Quantitative analyses were performed on images acquired from 3–4 fields per animal, randomly selected within the predefined region of interest (ROI) at the lesion core or perilesional region, as appropriate for each marker [35,41]. Measurements obtained at the field level were averaged within each animal to generate a single animal-level value, and each animal was treated as an independent experimental unit. Depending on the endpoint, immunofluorescence analyses included 4 to 6 animals per group, as indicated in the corresponding figure legends. The number of sections analyzed per animal for each endpoint is indicated in the relevant figure legends. Fluorescence signal intensity, reflecting target-specific antibody labeling, was quantified in a blinded manner using ImageJ (v1.54g). For representative images, tonal range, and image sharpness (smart sharpen, 0.9 pixels) were uniformly adjusted using Adobe Photoshop 2025 (Adobe Systems Inc., San Jose, CA, USA). To minimize bias, both image acquisition and analysis were randomized, and fluorescence thresholding parameters were applied prior to quantification.

### 2.12. Quantification and Statistical Analysis

Statistical analyses were performed in GraphPad Prism (v10.1.0). For BV2 in vitro datasets (cAMP, ARG1 densitometry, and p-p38 densitometry), groups were compared by one-way ANOVA followed by Bonferroni’s multiple-comparisons test. Longitudinal BBB scores were analyzed by two-way repeated-measures ANOVA (treatment × time) with multiplicity-adjusted post hoc testing [41]. Grid-walk footfall errors were evaluated across treatment groups at the 8-week post-injury time point using one-way ANOVA with Bonferroni correction (as applied in the Results section/figure analyses). Lesion cavity volume, gray matter area, and white matter sparing were quantified from serial transverse spinal cord sections using established morphometric methods, and immunofluorescence endpoints (e.g., ARG1, CD206, and p-p38 MAPK in Iba1^+^ myeloid cells) were quantified from confocal images acquired within predefined ROIs. Histological endpoints summarized per animal (total lesion cavity volume and gray matter area) and immunohistochemical intensity measures (ARG1 IR, CD206 IR, p-p38 MAPK^+^, Iba1^+^ cell percentages, GAP-43 IR) were analyzed by one-way ANOVA with Dunnett’s multiple-comparisons test versus SCI controls. For two-group comparisons (3D reconstructed cavity volume: SCI vs. combined treatment), an unpaired two-tailed Student’s t-test was used. Statistical significance was set at *p* < 0.05, with significance denoted as ns, * *p* < 0.05, ** *p* < 0.01, *** *p* < 0.001, and **** *p* < 0.0001. Data are presented as mean ± SEM unless otherwise indicated.

## 3. Results

### 3.1. PPF Enhances IL-4 Mediated Anti-Inflammatory Polarization of Microglia.

**Figure 1:**
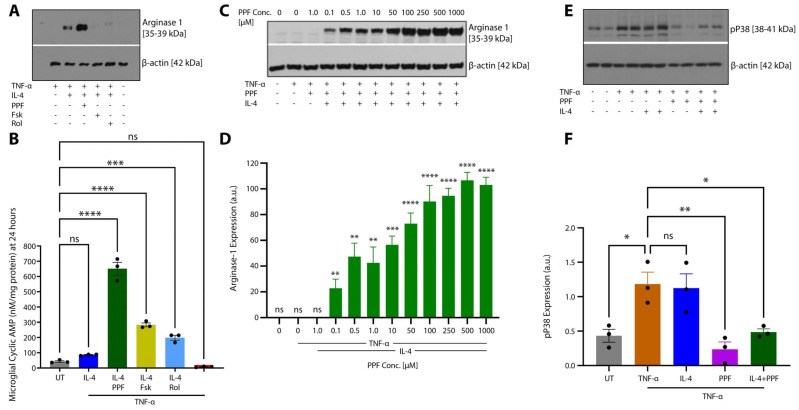
PPF potentiates IL-4-associated reparative signaling in TNF-α-challenged BV2 microglia and alters p38 phosphorylation. (**A**) Representative Western blot of ARG1(35–39 kDa) with β-actin (42 kDa) loading control in BV2 cells treated with TNF-α and the indicated combinations of IL-4 and PPF. Fsk and Rol are included as pharmacologic comparators that elevate cAMP signaling. (**B**) Intracellular cyclic AMP (cAMP) levels measured in untreated (UT) BV2 microglia or at 24 h under the treatment conditions shown. Bars represent mean ± SEM; dots represent individual biological replicates (*n* = 3 per condition). (**C**) Representative Western blot showing ARG1 expression across increasing concentrations of PPF (µM) in TNF-α-primed BV2 cells in the presence or absence of IL-4, as indicated; β-actin serves as the loading control. (**D**) Densitometric quantification of ARG1 from (**C**), normalized to β-actin and expressed in arbitrary units (a.u.). Bars represent mean ± SEM with individual replicates shown (*n* = 3 per condition). (**E**) Representative Western blot of p-p38 MAPK (p-p38; 38–41 kDa) with β-actin loading control in TNF-α-stimulated BV2 cells treated with IL-4 and/or PPF as indicated. (**F**) Densitometric quantification of p-p38 from (**E**), normalized to β-actin and expressed in a.u. Bars represent mean ± SEM; dots represent individual replicates (*n* = 3 per condition). For all quantified panels, statistical analysis was performed using one-way ANOVA followed by Bonferroni’s multiple-comparisons test. Significance is denoted as ns (not significant), * *p* < 0.05, ** *p* < 0.01, *** *p* < 0.001, **** *p* < 0.0001.

To evaluate the effects of PPF on microglial signaling and polarization-associated protein expression under inflammatory conditions, ARG1 protein expression was assessed by immunoblotting and quantified alongside intracellular cAMP accumulation and p38 MAPK phosphorylation (p-p38), with β-actin serving as a loading control throughout (Figure 1A,C,E). Under TNF-α stimulation, there was low level of detectable ARG1, whereas co-treatment with PPF in the presence of TNF-α + IL-4 resulted in a marked increase in ARG1 immunoreactivity (Figure 1A). In contrast, when TNF-α + IL-4-treated cells were exposed to forskolin (Fsk; adenylate cyclase activator) or to rolipram (Rol; Phosphodiesterase 4 [PDE4] inhibitor), ARG1 induction was comparatively weak or absent, indicating that PPF is substantially more effective at promoting ARG1 accumulation when cells were co-primed with IL-4 (Figure 1A).

Consistent with a strong second-messenger response, measurement of intracellular cAMP in untreated cells or at 24 h post-TNF-α priming showed low basal levels and no significant change with IL-4 alone (ns), whereas TNF-α + IL-4 plus PPF elicited a robust increase in intracellular cAMP concentrations (Figure 1B). Although Fsk and Rol elevated cAMP under TNF-α + IL-4 conditions, both responses were significantly smaller than the increase observed with PPF (Figure 1B), and TNF-α alone showed near-baseline levels of cAMP. A concentration response analysis further demonstrated that ARG1 induction by PPF was dose-dependent: in the presence of TNF-α, PPF alone (1.0 µM) did not significantly increase ARG1 (ns), whereas adding IL-4 revealed a progressive rise in ARG1 expression across increasing PPF concentrations (0.1–1000 µM), reaching maximal levels at higher doses (Figure 1C,D). Finally, analysis of p-p38 activation showed that TNF-α increased p-p38 relative to untreated controls (Figure 1E), while IL-4 did not significantly alter TNF-α driven p-p38 expression (ns) (Figure 1E,F). In contrast, PPF significantly suppressed p-p38 in TNF-α primed microglia, and the combination of IL-4 + PPF also reduced p-p38 relative to TNF-α alone (Figure 1E,F). Collectively, these data indicate that PPF strongly elevates microglial cAMP, drives a dose-dependent increase in ARG1 expression under TNF-α + IL-4 co-stimulation, and attenuates TNF-α-associated p38 phosphorylation.

### 3.2. Combined Treatment Improves Gross and Fine Locomotor Recovery Following SCI

**Figure 2:**
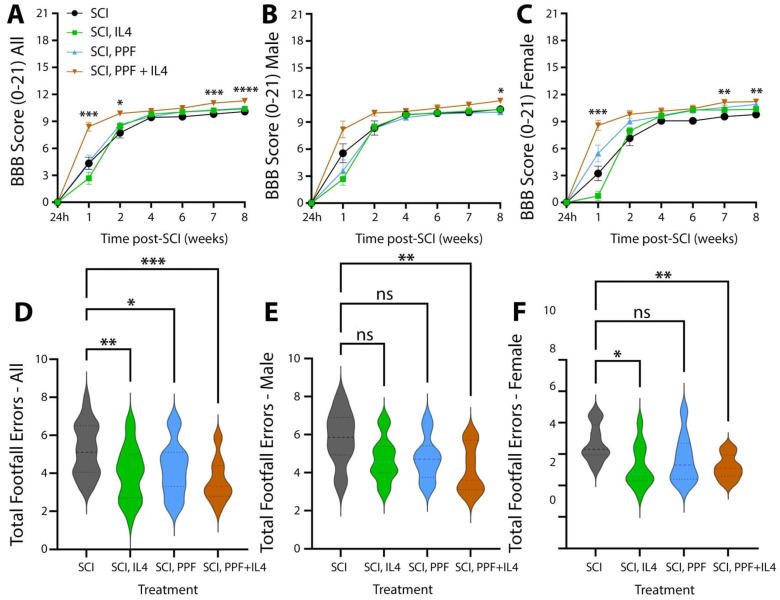
Combined PPF and IL-4 treatment improves gross and fine locomotor recovery after SCI. Adult Fischer rats received daily intraperitoneal injections of vehicle, PPF, IL-4, or combined PPF + IL-4, beginning within 1 h after moderate thoracic contusion SCI and continuing for 2 weeks post-injury. (**A**–**C**) Open-field locomotor recovery was assessed using the Basso, Beattie, and Bresnahan (BBB) score (0–21) from 24 h to 8 weeks post-SCI in the combined cohort (**A**), males (**B**), and females (**C**). Data are presented as mean ± SEM. Combined PPF + IL-4 treatment significantly improved BBB scores relative to SCI and monotherapy groups at multiple time points, with sex-specific differences in the magnitude and timing of recovery. (**D**–**F**) Fine locomotor recovery was assessed at 8 weeks post-SCI using the grid-walk task and is shown as total hindlimb footfall errors for the combined cohort (**D**), males (**E**), and females (**F**). Violin plots show the distribution, with dashed lines indicating the median and quartiles. Combined treatment reduced footfall errors relative to SCI, with sex-dependent differences in significance across treatment groups. Sample sizes were SCI (*n* = 25; 13 females, 12 males), SCI + PPF (*n* = 19; 9 females, 10 males), SCI + IL-4 (*n* = 23; 11 females, 12 males), and SCI + PPF + IL-4 (*n* = 25; 14 females, 11 males). Group sizes were different due to predefined exclusions, including deviation from target injury parameters. BBB post hoc comparisons were analyzed using Tukey’s multiple-comparisons test. Statistical significance is indicated as *p* < 0.05 (*)*, p <* 0.01 (**), *p <* 0.001 (***), and *p* < 0.0001 (****); ns, not significant.

Locomotor recovery was evaluated longitudinally using the BBB open-field locomotor score and at the chronic endpoint using the grid-walk task. In the combined cohort, all groups showed progressive improvement in BBB scores over the 8-week post-injury period; however, animals treated with combined PPF + IL-4 exhibited superior gross locomotor recovery relative to SCI controls and both monotherapy groups at multiple time points (Figure 2A; Supplementary Table S1). Specifically, the combined treatment group showed significantly higher BBB scores than the SCI group at weeks 1, 2, 7, and 8. BBB scores were also significantly higher in the combined treatment group than in the SCI + IL-4 group at weeks 1, 2, 7, and 8, and higher than in the SCI + PPF group at weeks 1, 2, 7, and 8. These findings indicate that the combination of PPF and IL-4 produced a greater improvement in gross locomotor recovery than either treatment alone.

To determine whether treatment responses differed by sex, BBB outcomes were analyzed separately in males and females. In males, the combined PPF + IL-4 treatment group showed a modest but significant improvement in BBB score at week 8 relative to SCI controls, whereas no significant differences were detected between the combined treatment group and either monotherapy group at any time point examined (Figure 2B; Supplementary Table S1). In contrast, in females, combined treatment produced a more robust functional benefit (Figure 2C; Supplementary Table S1). Female rats receiving PPF + IL-4 had significantly higher BBB scores than SCI controls at weeks 1, 7, and 8. In addition, at weeks 7 and 8, BBB scores in the combined treatment group were significantly higher than those in the SCI + IL-4 group. No significant differences were observed between the combined treatment and SCI + PPF groups in females.

Fine locomotor function was assessed at 8 weeks post-injury using the grid-walk task by quantifying total hindlimb footfall errors. All treatment groups showed statistically significant fewer footfall errors than SCI controls, with the greatest reduction observed in the PPF + IL-4 group (Figure 2D). In the male cohort, only the combined treatment group showed a significant reduction in footfall errors relative to SCI controls, whereas neither monotherapy group differed significantly from SCI (Figure 2E). In females, both the SCI + IL-4 group and the combined PPF + IL-4 group exhibited significantly fewer footfall errors than SCI controls, while the SCI + PPF group did not differ significantly from SCI (Figure 2F). Collectively, these findings demonstrate that combined PPF + IL-4 treatment improves both gross and fine locomotor recovery after thoracic contusion SCI, with the most pronounced benefit observed in females.

### 3.3. Combined Treatment Reveals a Trend for Improved Preservation of Spinal Cord Architecture and Myelin Integrity

**Figure 3:**
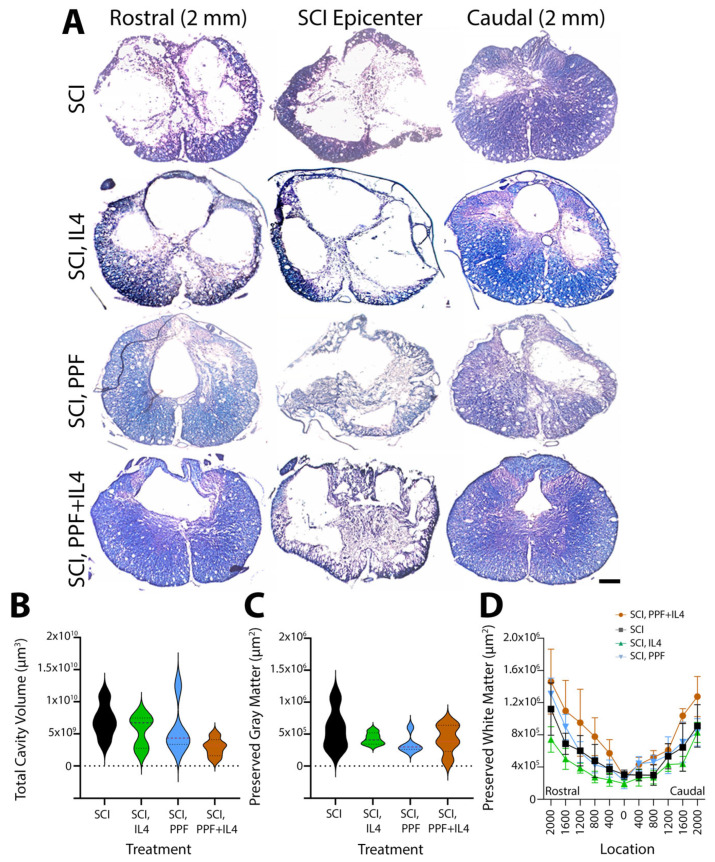
Histological assessment of lesion pathology and tissue sparing after SCI shows a trend towards improved preservation of white matter with combined PPF and IL-4 treatment. (**A**) Representative transverse spinal cord sections stained with hematoxylin, eosin and a myelin-sensitive histological stain (LFB based myelin staining) to visualize white matter integrity and lesion-associated cavitation. Images are shown for three rostro-caudal levels relative to the injury: rostral (2 mm), SCI epicenter, and caudal (2 mm). Rows depict treatment groups: SCI (vehicle), SCI + IL-4, SCI + PPF, and SCI + PPF + IL-4. Loss of staining and tissue discontinuity at the epicenter indicate lesion core/cavitation, whereas preserved dark/blue-stained regions reflect spared myelinated white matter. Scale bar shown in the lower-right image (applies to all sections). (**B**) Quantification of total lesion cavity volume (µm^3^) across treatment groups, displayed as violin plots representing the distribution of values across animals; internal dashed lines indicate central tendency (as defined in Methods) and the horizontal dotted line marks 0. (**C**) Quantification of preserved gray matter area (µm^2^) across groups (violin plots; plotting conventions as in (**B**)), determined by morphometric analysis of serial cross-sections spanning the lesion. (**D**) White matter sparing plotted as preserved white matter area (µm^2^) as a function of rostro-caudal distance from the epicenter (location axis; negative values rostral and positive values caudal, as labeled). Points represent mean ± SEM for each position for SCI, SCI + IL-4, SCI + PPF, and SCI + PPF + IL-4 groups. The plotted values show the distribution of preserved white matter area across the lesion span for each treatment group. Each group includes *n* = 5 animals. Data are presented as mean ± SEM. For violin plots, dashed/dotted lines indicate the median and quartiles as shown. Statistical analysis was performed using one-way ANOVA with Dunnett’s multiple-comparisons test; significance is indicated in the figure where applicable. Scale bar = 400 µm.

Histological analyses were performed to evaluate tissue preservation at eight weeks following SCI. Vehicle-treated animals exhibited extensive cavitation with disruption of spinal cord architecture and severe loss of myelinated axons as revealed by H&E and LFB staining (Figure 3A). Animals treated with IL-4 or PPF monotherapy produced modest reductions in lesion size with partial gray and white matter preservation. Combined IL-4 + PPF was associated with reduced cavitation and qualitative preservation of spinal cord architecture, with a trend toward greater white matter sparing along the rostro-caudal axis. although these effects did not reach statistical significance in the rostro-caudal analysis performed (Figure 3B–D). Quantitatively, the IL-4 + PPF combination exhibited reduced total lesion cavity volume (Figure 3B) and increased preserved gray matter area compared with vehicle and monotherapy groups (Figure 3C). Quantitative assessment of spared white matter showed a trend toward greater preservation at all distances from the injury epicenter in animals receiving the combined therapy versus untreated SCI controls, although these differences did not reach statistical significance (Figure 3D).

### 3.4. Propentofylline Treatment Reduces Degraded Myelin in the Perilesional Spinal Cord Region After SCI

**Figure 4:**
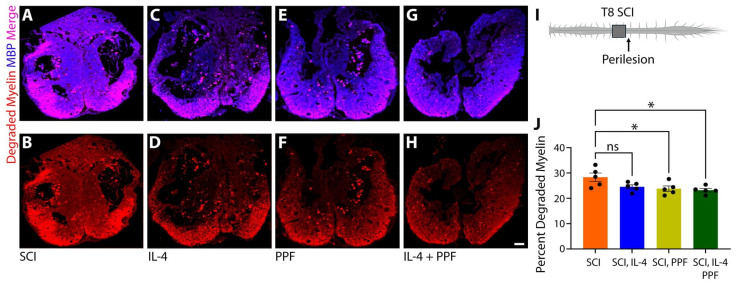
Combined PPF + IL-4 treatment reduces degraded myelin in the perilesional spinal cord after SCI. Adult Fischer rats received daily intraperitoneal injections of vehicle, PPF, IL-4, or combined PPF + IL-4, beginning within 1 h after moderate thoracic contusion SCI and continuing for 2 weeks post-injury. (**A**–**H**) Representative transverse spinal cord sections from the perilesional region located 800 μm caudal to the injury epicenter are shown for SCI (**A**,**B**), SCI + IL-4 (**C**,**D**), SCI + PPF (**E**,**F**), and SCI + PPF + IL-4 (**G**,**H**). Merged images (**A**,**C**,**E**,**G**) show degraded myelin (magenta) and myelin basic protein (MBP; blue), and the corresponding single-channel images (**B**,**D**,**F**,**H**) show degraded myelin signal alone. (**I**) Schematic showing the perilesional region used for histological analysis. (**J**) Quantification of the percentage of degraded myelin in the perilesional spinal cord across treatment groups. Each group includes *n* = 5 animals. For immunofluorescence analyses, 3–4 fields per animal were acquired from the predefined ROI and averaged to generate one value per animal; the animal was treated as the independent experimental unit. Data are presented as mean ± SEM. Statistical analysis was performed using one-way ANOVA with Dunnett’s multiple-comparisons test; significance is indicated in the figure where applicable. The percentage of degraded myelin was lower in the SCI + PPF and SCI + PPF + IL-4 groups than in the SCI group, whereas the SCI + IL-4 group did not significantly differ from SCI. Statistical significance is indicated in the figure where applicable (* *p* < 0.05; ns, not significant). Scale bar = 50 µm.

To determine whether IL-4 and/or PPF treatment influenced myelin pathology after SCI, degraded myelin was quantified in the perilesional region located 800 µm caudal to the injury epicenter. Representative transverse spinal cord sections illustrate degraded myelin labeling (magenta) and myelin basic protein (MBP; blue) across treatment groups (Figure 4A–H). Vehicle-treated SCI animals exhibited prominent degraded myelin signal within the perilesional white matter, consistent with persistent myelin degeneration two months after injury.

Quantitative analysis revealed that treatment with PPF significantly reduced the percentage of degraded myelin compared with SCI controls (Figure 4J). A similar reduction was observed in animals receiving the combined PPF + IL-4 treatment. In contrast, the IL-4 monotherapy did not significantly alter the extent of degraded myelin relative to SCI controls. Notably, the magnitude of myelin preservation in the combined PPF + IL-4 group was comparable to that observed with PPF alone.

### 3.5. Combined Treatment Reduces Lesion Cavity Volume in Three-Dimensional Reconstructions

**Figure 5:**
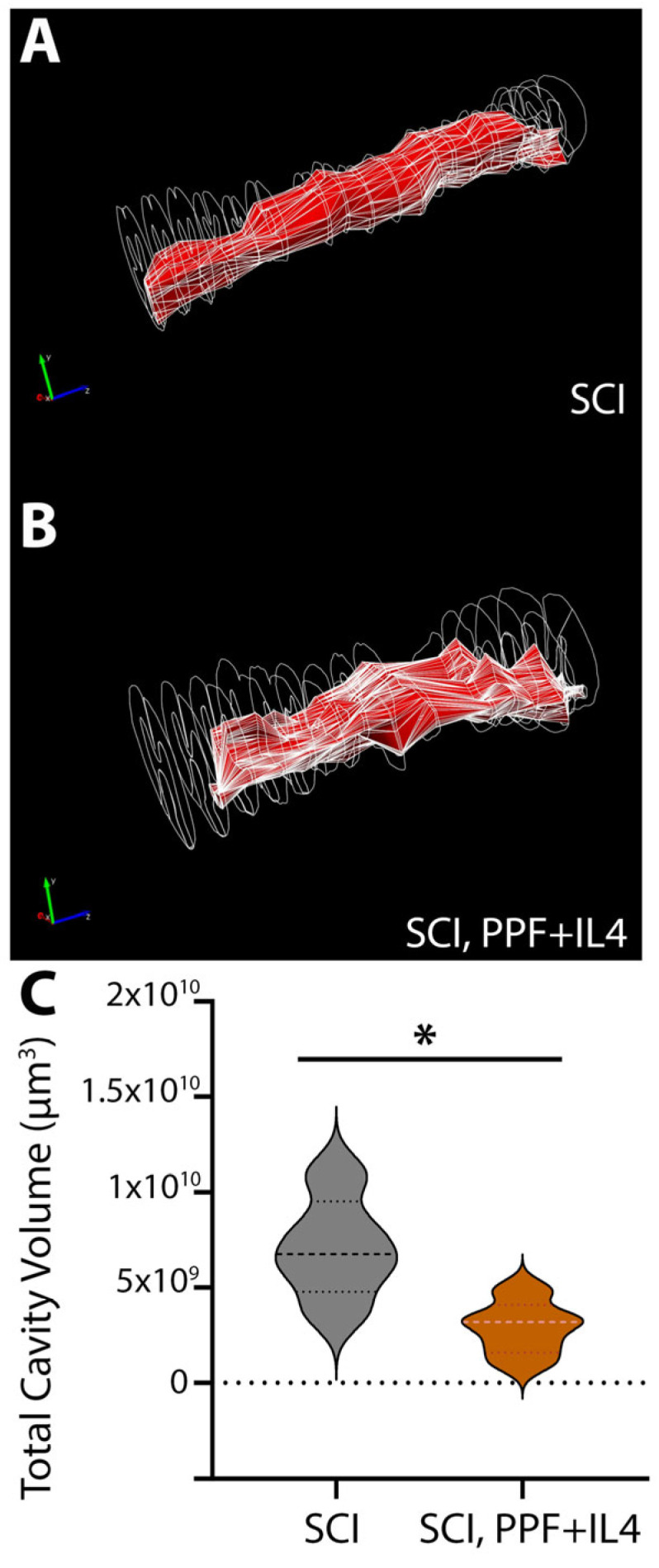
Three-dimensional reconstruction of lesion cavitation reveals reduced total cavity volume with combined PPF and IL-4 treatment after SCI. (**A**,**B**) Representative 3D renderings of the lesion cavity generated from serial transverse spinal cord sections spanning the injury region. The lesion cavity is segmented and displayed in red, with the outlines of individual section contours/registration shown in white/gray to illustrate the reconstructed volume along the rostro-caudal axis. Renderings are shown for SCI (vehicle) (**A**) and SCI + PPF + IL-4 (**B**). Orientation axes are indicated in the lower-left corner. (**C**) Quantification of total lesion cavity volume (µm^3^) comparing SCI and SCI with PPF and IL-4 groups. Data are presented as violin plots showing the distribution of individual animal values; internal dashed lines indicate central tendency (as defined in Section 2 Methods) and the horizontal dotted line marks 0. Total cavity volume was lower in the SCI + PPF + IL-4 group than in the SCI group. Both groups include *n* = 5 animals. Statistical significance is indicated by * *p* < 0.05.

To further assess lesion morphology, three-dimensional reconstructions of lesion cavities were generated from serial transverse spinal cord sections (Figure 5). Vehicle-treated animals displayed large, elongated cavities extending across multiple spinal cord segments (Figure 5A). In contrast, animals treated with combined PPF and IL-4 exhibited smaller, more compact lesion cavities with a reduced rostro-caudal extent (Figure 5B). Quantitative volumetric analysis confirmed a significant reduction in total cavity volume in the combined treatment group compared to vehicle controls (Figure 5C).

### 3.6. Combined IL-4 and PPF Elevate Lesion ARG1 After T8 Spinal Cord Injury

**Figure 6:**
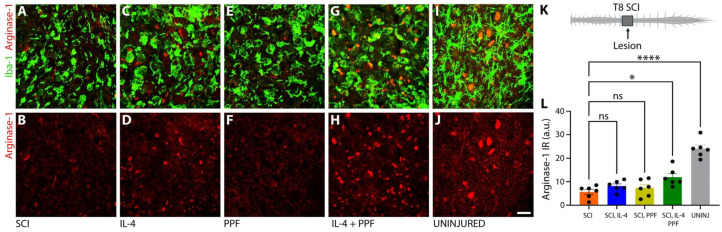
PPF and IL-4 cooperatively increase ARG1 immunoreactivity in lesion-associated Iba1^+^ myeloid cells after T8 SCI. (**A**–**J**) Representative immunofluorescence images of transverse spinal cord sections collected from the injury epicenter 2 months after T8 spinal cord contusion. Microglia/macrophages were labeled with Iba1 (green), and the reparative marker ARG1 was labeled in red. Merged images are shown in (**A**,**C**,**E**,**G**,**I**), and the corresponding ARG1 channel alone is shown in (**B**,**D**,**F**,**H**,**J**) for the following groups: SCI (**A**,**B**), SCI + IL-4 (**C**,**D**), SCI + PPF (**E**,**F**), SCI + IL-4 + PPF (**G**,**H**), and uninjured (**I**,**J**). Scale bar = 10 µm. (**K**) Schematic illustrating the T8 SCI level and the lesion-centered region used for image acquisition and quantification. (**L**) Quantification of ARG1 immunoreactivity (IR; a.u.) within the lesion region at the injury epicenter. ARG1 IR differed across groups as indicated in the figure. Bars represent mean ± SEM, and dots represent individual animals (*n* = 5 per group). For immunofluorescence analyses, 3–4 fields per animal were acquired from the predefined ROI and averaged to generate one value per animal; the animal was treated as the independent experimental unit. Statistical analysis was performed using one-way ANOVA followed by Dunnett’s multiple-comparisons test. Significance is indicated as shown in the figure: ns, not significant; * *p* < 0.05; **** *p* < 0.0001.

To determine whether IL-4 and/or PPF promote an ARG1-associated reparative phenotype in lesion-associated myeloid cells, ARG1 immunoreactivity was quantified at the T8 SCI epicenter two months after contusion (Figure 6A–L). Representative confocal images show dense accumulation of Iba1^+^ microglia/macrophages throughout the lesion region in all injured groups (Iba1, green; Figure 6A,C,E,G). In SCI controls, ARG1 labeling (red) was sparse, appearing as scattered ARG1^+^ cellular profiles within the lesion core (Figure 6A,B). Treatment with IL-4 alone or PPF alone produced only modest qualitative increases in ARG1 signal compared with SCI controls, with relatively limited numbers of ARG1^+^ cells and overall labeling intensity remaining comparable to the vehicle-treated SCI group (Figure 6C–F). In contrast, combined IL-4 + PPF treatment produced a clear increase in ARG1 immunoreactivity within the lesion region, with more numerous and brighter ARG1^+^ cellular profiles interspersed among Iba1^+^ myeloid cells (Figure 6G,H). As expected, uninjured spinal cord tissue displayed robust ARG1 labeling distributed among resident Iba1^+^ cells and other cellular elements, reflecting the baseline ARG1 signal present in intact spinal cord tissue (Figure 6I,J).

Quantitative analysis of ARG1 immunoreactivity confirmed these observations (Figure 6L). Neither IL-4 alone nor PPF alone significantly increased ARG1 immunoreactivity compared with SCI controls (both comparisons ns). In contrast, the combined IL-4 + PPF treatment significantly elevated ARG1 immunoreactivity relative to SCI controls (*p* < 0.05). Notably, ARG1 signal in uninjured spinal cord was substantially higher than that observed in injured groups and was significantly greater than the combined treatment group (*** *p* < 0.0001), highlighting the persistent reduction in ARG1-associated signaling following injury. Because histological endpoints were assessed in a predefined subset of animals (*n* = 5 per group), ARG1 immunoreactivity analyses were performed on pooled male and female cohorts. Subgroup sizes were insufficient to support statistically robust sex-disaggregated comparisons; therefore, these analyses should be interpreted as reflecting overall treatment-associated trends across a mixed-sex cohort.

Collectively, these findings demonstrate that IL-4 and PPF act cooperatively to enhance ARG1 expression within lesion-associated Iba1^+^ myeloid cells after T8 SCI. However, even with combined treatment, ARG1 levels remain below those observed in uninjured spinal cord, indicating that injury-induced alterations in myeloid activation state are only partially restored by this therapeutic intervention.

### 3.7. PPF Suppresses Chronic p38 MAPK Activation in Lesion-Associated Iba1^+^ Myeloid Cells After SCI and Maintains Low p-p38 Signaling When Combined with IL-4

**Figure 7:**
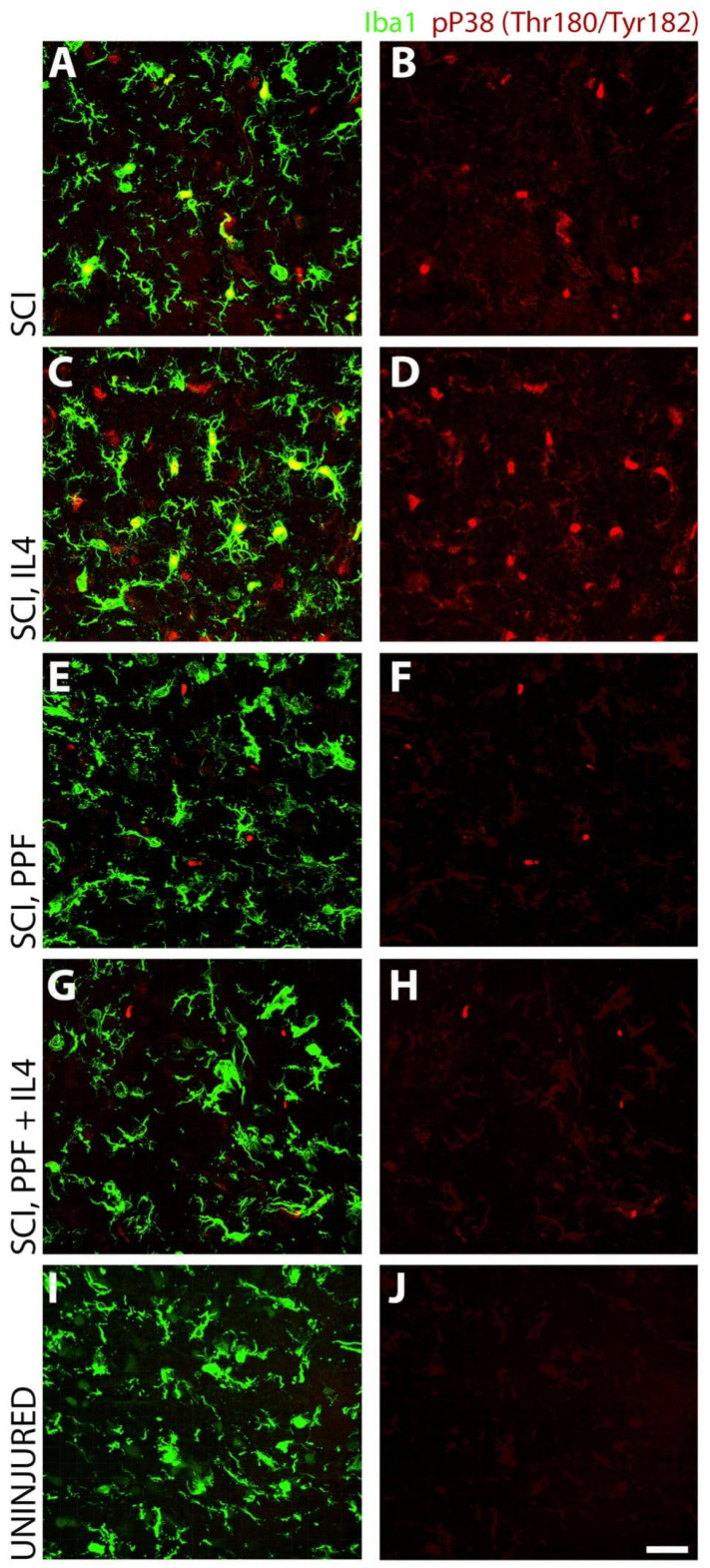
Combined IL-4 + PPF treatment suppresses lesion-associated pP38 (Thr180/Tyr182) immunoreactivity in Iba1+ myeloid cells after SCI. (**A**–**J**) Representative immunofluorescence micrographs from transverse spinal cord sections collected 2 months after T8 spinal cord contusion. Iba1+ myeloid cells are labeled in green and phosphorylated p38 MAPK [pP38 (Thr180/Tyr182)] is labeled in red. (**A**,**C**,**E**,**G**,**I**) show merged channels, and (**B**,**D**,**F**,**H**,**J**) show the corresponding pP38-only channel for each group: SCI (**A**,**B**), SCI + IL-4 (**C**,**D**), SCI + PPF (**E**,**F**), SCI + IL-4 + PPF (**G**,**H**), and uninjured (**I**,**J**). The images show prominent pP38 immunoreactivity in lesion-associated Iba1+ cells in the SCI group, lower apparent pP38 signal in the treatment groups, and low basal staining in the uninjured spinal cord. Scale bar = 10 μm.

**Figure 8:**
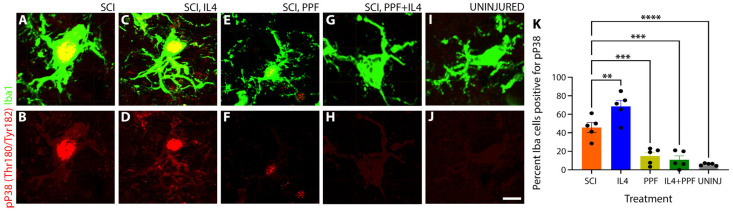
PPF suppresses p38 MAPK activation in Iba1+ myeloid cells after SCI and maintains low p-p38 signal when combined with IL-4. (**A**–**J**) Representative high-magnification confocal images from spinal cord sections collected 2 months after thoracic SCI showing Iba1+ myeloid cells (green) and activated p38 MAPK detected with an antibody against p-p38 (Thr180/Tyr182) (red). Groups shown are SCI (**A**,**B**), SCI + IL-4 (**C**, **D**), SCI + PPF (**E**, **F**), SCI + PPF + IL-4 (**G**,**H**), and uninjured (**I**,**J**). Panels (**A**,**C**,**E**,**G**,**I**) show the Iba1 channel for each group, highlighting myeloid cell morphology, whereas panels (**B**,**D**,**F,H**,**J**) show the corresponding p-p38 channel from the same fields. Compared with vehicle-treated SCI, the proportion of p-p38-positive Iba1+ cells differed across treatment groups, with lower p-p38 labeling in the PPF and PPF + IL-4 groups, as indicated in the figure. Low basal p-p38 staining was observed in the uninjured spinal cord. (**K**) Quantification of the percentage of Iba1+ cells positive for p-p38 (Thr180/Tyr182) across treatment groups. Bars represent mean ± SEM and dots represent individual animals. Each group includes *n* = 5 animals. For immunofluorescence analyses, a minimum of three fields per animal were acquired from the predefined ROI and averaged to generate one value per animal; the animal was treated as the independent experimental unit. Statistical significance was assessed using one-way ANOVA with Dunnett’s multiple-comparisons test and is indicated in the figure (** *p* < 0.01, *** *p* < 0.001, **** *p* < 0.0001). Scale bar = 5 µm.

To assess the effects of treatment on inflammatory signaling in lesion-associated Iba1^+^ myeloid cells, spinal cord sections collected 2 months after T8 SCI were immunolabeled for Iba1 and phosphorylated p38 MAPK [p-p38 (Thr180/Tyr182)] (Figure 7 and Figure 8). In low-magnification sections, vehicle-treated SCI animals exhibited prominent p-p38 immunoreactivity within lesion-associated Iba1^+^ cells, consistent with persistent inflammatory activation in the chronic injury environment (Figure 7A,B). MAPK p-p38 labeling remained readily detectable in the IL-4-treated group (Figure 7C,D), whereas PPF treatment markedly reduced p-p38 signal (Figure 7E,F). The combined IL-4 + PPF group similarly displayed low p-p38 immunoreactivity (Figure 7G,H). In contrast, uninjured spinal cord showed minimal basal p-p38 staining (Figure 7I,J), indicating that SCI is associated with sustained activation of p38 MAPK in myeloid cells that is attenuated by PPF-containing treatment regimens.

High-magnification confocal imaging further resolved p-p38 localization within individual Iba1^+^ myeloid cells (Figure 8A–J). In vehicle-treated SCI animals, lesion-associated Iba1^+^ cells showed robust p-p38 signal, frequently concentrated in the cell body and consistent with ongoing intracellular stress and inflammatory signaling (Figure 8A,B). IL-4 monotherapy was associated with a high proportion of p-p38-positive Iba1^+^ cells, with many cells retaining strong p-p38 labeling (Figure 8C,D). In contrast, PPF monotherapy markedly attenuated p-p38 immunoreactivity within Iba1^+^ cells (Figure 8E,F), and the IL-4 + PPF group showed a similarly low level of p-p38 staining (Figure 8G,H). Uninjured tissue exhibited low basal p-p38 immunoreactivity, with only rare Iba1^+^ cells positive for p-p38 (Figure 8I,J).

Quantification of the percentage of Iba1^+^ cells positive for p-p38 confirmed these observations (Figure 8K). Relative to SCI controls, IL-4 treatment significantly increased the proportion of p-p38-positive Iba1^+^ cells, whereas both PPF monotherapy and combined IL-4 + PPF treatment significantly reduced the percentage of p-p38-positive Iba1^+^ cells. The uninjured group exhibited the lowest proportion of p-p38-positive Iba1^+^ cells and differed markedly from the injured groups, consistent with low basal p38 MAPK activity in the intact spinal cord. Notably, the combined IL-4 + PPF group was not detectably different from PPF monotherapy, indicating that suppression of chronic p38 MAPK activation in lesion-associated myeloid cells is driven primarily by PPF. Together, these findings identify PPF as the principal mediator of reduced chronic p38 MAPK signaling after SCI and show that it prevents the elevated p-p38 profile observed with IL-4 treatment alone, maintaining myeloid p38 activation at levels closer to those observed in uninjured tissue.

### 3.8. IL-4 and PPF Suppress Lesion-Associated p65 NFκB Immunoreactivity After T8 Spinal Cord Injury

**Figure 9:**
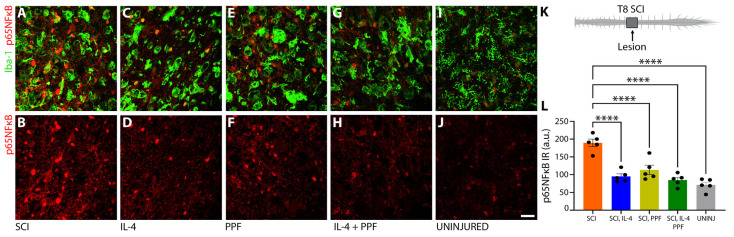
Combined IL-4 + PPF treatment suppresses lesion-associated p65 NFκB immunoreactivity after T8 SCI. (**A**–**J**) Representative immunofluorescence micrographs from transverse spinal cord sections collected from the injury epicenter at 2 months after T8 spinal cord contusion. Iba1+ myeloid cells are labeled in green and p65 NFκB is labeled in red. (**A**,**C**,**E**,**G**,**I**) show merged channels, and (**B**,**D**,**F**,**H**,**J**) show the corresponding p65 NFκB-only channel for each group: SCI (**A**,**B**), SCI + IL-4 (**C**,**D**), SCI + PPF (**E**,**F**), SCI + IL-4 + PPF (**G**,**H**), and uninjured (**I**,**J**). (**K**) Schematic depicting the T8 SCI level and lesion-centered region used for imaging and quantification. (**L**) Quantification of p65 NFκB immunoreactivity (IR; a.u.) within the lesion region at 2 months post-SCI. Bars represent mean ± SEM and dots represent individual animals. For immunofluorescence quantification, 3–4 fields per animal were acquired from the lesion-centered ROI and averaged to generate one value per animal. Each group included *n* = 5 animals. p65 NFκB IR was lower in the SCI + IL-4, SCI + PPF, SCI + IL-4 + PPF, and uninjured groups than in the SCI group. Statistical analysis was performed using one-way ANOVA with Dunnett’s multiple-comparisons test. Significance is denoted as **** *p* < 0.0001. Scale bar = 10 μm.

To determine whether treatment modulates inflammatory transcriptional signaling after SCI, immunofluorescence labeling for the NF-κB subunit p65 was performed in transverse spinal cord sections collected from the lesion epicenter at 2 months following T8 contusion injury (Figure 9A–J). Iba1 immunolabeling was used to identify lesion-associated myeloid cells.

Vehicle-treated SCI animals exhibited robust p65 NFκB immunoreactivity within the lesion region, with numerous Iba1^+^ cells displaying strong nuclear and cytoplasmic p65 NFκB signal (Figure 9A,B), consistent with persistent inflammatory activation at the chronic stage after injury. Treatment with IL-4 alone was associated with a marked reduction in p65 NFκB signal within the lesion area compared with SCI controls (Figure 9C,D). Similarly, PPF monotherapy produced a noticeable attenuation of p65 NFκB. 

NFκB immunoreactivity in the lesion-associated cellular population (Figure 9E,F). Animals receiving the combined IL-4 + PPF treatment displayed the lowest apparent p65 NFκB signal among injured groups, with reduced red fluorescence intensity throughout the lesion region (Figure 9G,H). In contrast, uninjured spinal cord tissue exhibited minimal p65 NFκB immunoreactivity (Figure 9I,J).

Quantitative analysis confirmed these observations (Figure 9L). p65 NFκB immunoreactivity within the lesion region was significantly reduced in the SCI + IL-4, SCI + PPF, SCI + IL-4 + PPF, and uninjured groups relative to vehicle-treated SCI animals (*p* < 0.0001 for all comparisons). Together, these findings indicate that IL-4 and PPF treatments markedly suppress NF-κB-associated inflammatory signaling in the chronically injured spinal cord, with the combined treatment producing particularly strong suppression of lesion-associated p65 NFκB activity.

### 3.9. Combined PPF and IL-4 Treatment Promotes Sustained Reparative Lesion-Associated Myeloid Cell Polarization After SCI

**Figure 10:**
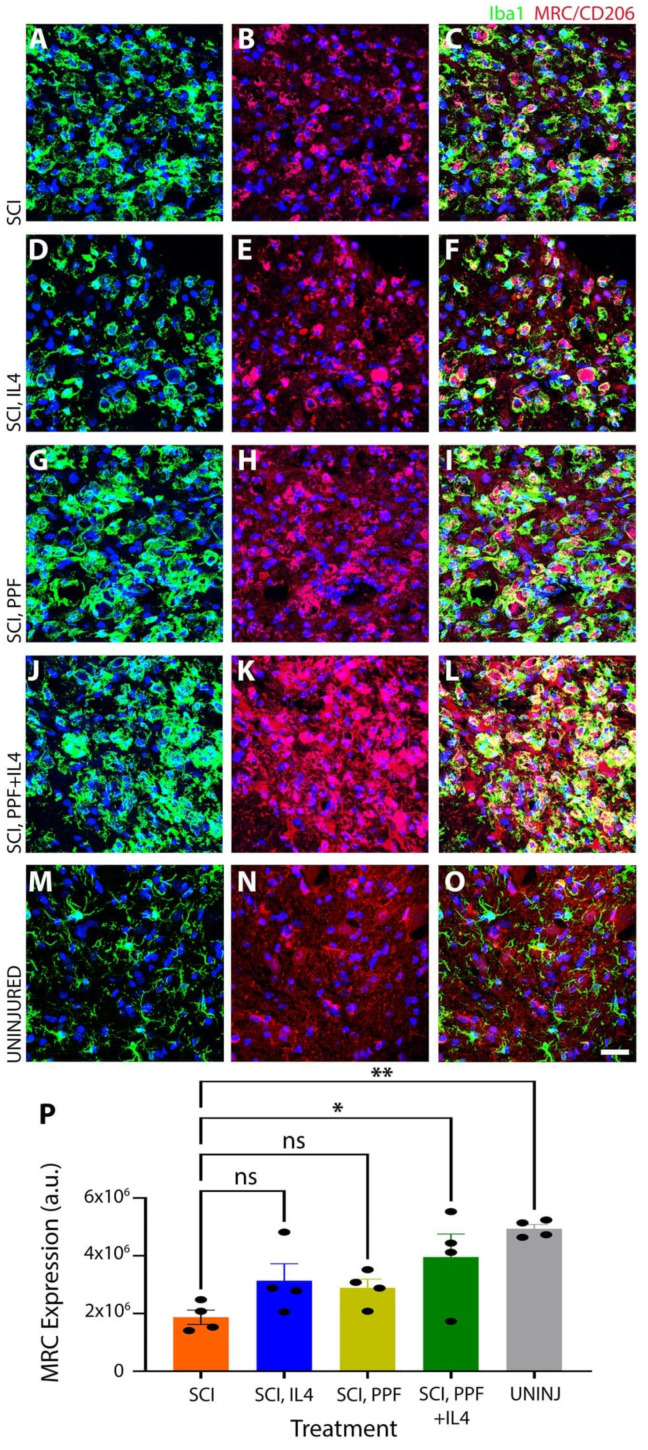
Combined PPF and IL-4 treatment promote sustained reparative lesion-associated Iba1+ myeloid cell polarization in the injured spinal cord. (**A**–**O**) Representative confocal images of spinal cord sections collected from the lesion center at 2 months after SCI under the indicated treatment conditions: SCI (**A**–**C**), SCI + IL-4 (**D**–**F**), SCI + PPF (**G**–**I**), SCI + PPF + IL-4 (**J**–**L**), and uninjured (**M**–**O**). Iba1+ myeloid cells are labeled in green, the mannose receptor MRC/CD206 is labeled in red, and nuclei are counterstained with Hoechst (blue). For each group, single-channel images are shown for Iba1 (**A**,**D**,**G**,**J**,**M**) and MRC/CD206 (**B**,**E**,**H**,**K**,**N**) with the corresponding merged images (**C**,**F**,**I**,**L**,**O**), illustrating the spatial relationship between Iba1+ myeloid cells and MRC/CD206 signal within the lesion microenvironment. (**P**) Quantification of MRC/CD206 expression (arbitrary units, a.u.) within the analyzed region. Bars represent mean ± SEM and dots represent individual animals. For immunofluorescence quantification, a minimum of three fields per animal were acquired from the lesion-centered ROI and averaged within animal. Each group included *n* = 4 animals. Compared with SCI, SCI + PPF + IL-4 showed increased MRC/CD206 immunoreactivity, whereas SCI + IL-4 and SCI + PPF were not significantly different from SCI. MRC/CD206 expression in the uninjured group was also significantly higher than in SCI. Statistical significance is indicated in the figure and was determined using one-way ANOVA with Dunnett’s multiple-comparisons test (* *p* < 0.05, ** *p* < 0.01; ns, not significant). Scale bar = 10 μm.

To determine whether the reduction in inflammatory signaling was accompanied by a shift toward a reparative myeloid cell phenotype, expression of the mannose receptor MRC/CD206 was evaluated in spinal cord sections collected from the lesion center at 2 months after SCI (Figure 10A–O). Iba1 immunolabeling was used to identify lesion-associated myeloid cells.

Vehicle-treated SCI animals exhibited relatively low MRC/CD206 immunoreactivity within the lesion region (Figure 10A–C). Animals treated with IL-4 alone showed a modest increase in MRC/CD206 signal compared with SCI controls, although this effect was limited (Figure 10D–F). Similarly, PPF monotherapy produced only a modest elevation in MRC/CD206 immunoreactivity relative to SCI (Figure 10G–I). In contrast, the combined PPF + IL-4 treatment resulted in a more pronounced increase in MRC/CD206 labeling within the lesion microenvironment (Figure 10J–L). As expected, the uninjured spinal cord also displayed higher MRC/CD206 expression than the injured SCI control group (Figure 10M–O).

Quantitative analysis confirmed that MRC/CD206 expression was significantly increased in the SCI + PPF + IL-4 group compared with SCI controls, whereas the SCI + IL-4 and SCI + PPF groups were not significantly different from SCI (Figure 10P). MRC/CD206 expression was also significantly higher in the uninjured group than in SCI. Together, these findings indicate that while IL-4 or PPF alone were insufficient to significantly enhance reparative lesion-associated myeloid polarization at this chronic time point, their combined administration promoted a sustained increase in MRC/CD206-associated reparative signaling after SCI.

### 3.10. Augmented GAP-43 Immunoreactivity in Perilesional Spinal Tissue Following IL-4 or IL-4 and Propentofylline Treatment at 2 Months After T8 SCI

**Figure 11:**
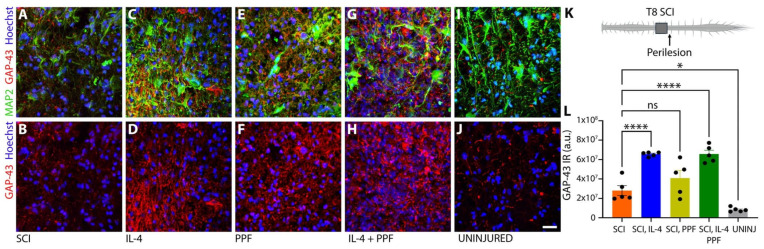
IL-4 or combined IL-4 + PPF treatment increases GAP43 immunoreactivity in the perilesional region 2 months after T8 SCI**.** (**A**–**J**) Representative immunofluorescence images from transverse spinal cord sections collected 2 months after T8 spinal cord contusion and acquired from the perilesional spinal cord region. Neuronal processes are labeled with MAP2 (green), GAP43 (red), and nuclei with Hoechst (blue). (**A**,**C**,**E**,**G**,**I**) show merged channels (MAP2 + GAP43 + Hoechst), and (**B**,**D**,**F**,**H**,**J**) show the corresponding GAP43-only channel for each group: SCI (**A**,**B**), SCI + IL-4 (**C**,**D**), SCI + PPF (**E**,**F**), SCI + IL-4 + PPF (**G**,**H**), and uninjured (**I**,**J**). (**K**) Schematic depicting the T8 SCI level and the perilesional region used for image acquisition and quantification. (**L**) Quantification of GAP43 immunoreactivity (IR; a.u.) in the perilesional region. For immunofluorescence quantification, 3–4 fields per animal were acquired from the perilesional ROI and averaged to generate one value per animal. Each group included *n* = 5 animals. Bars represent mean ± SEM and dots represent individual animals. GAP43 IR was higher in the SCI + IL-4 and SCI + IL-4 + PPF groups than in the SCI group, whereas the SCI + PPF group did not significantly differ from SCI. GAP43 IR in the uninjured group was lower than that observed in the SCI + IL-4 and SCI + IL-4 + PPF groups. Statistical analysis was performed using one-way ANOVA with Dunnett’s multiple-comparisons test. Significance is denoted as ns, * *p* < 0.05, and **** *p* < 0.0001. Scale bar = 10 μm.

To determine whether the treatment enhanced markers associated with axonal growth and structural plasticity in spared tissue adjacent to the lesion, GAP43 immunoreactivity was assessed in the perilesional spinal cord region at 2 months after T8 SCI (Figure 11A–L). MAP2 immunolabeling was used to visualize neuronal processes, while GAP43 labeling was used to assess growth-associated neuronal remodeling.

In vehicle-treated SCI animals, GAP43 immunoreactivity in the perilesional region was relatively low, with comparatively sparse and less intense red labeling distributed within the surrounding tissue (Figure 11A,B). In contrast, animals treated with IL-4 alone exhibited a marked increase in GAP43 signal, with more abundant and brighter GAP43-positive processes in the perilesional parenchyma (Figure 11C, D). PPF monotherapy showed a more variable and comparatively modest pattern of GAP43 labeling, with a detectable signal but no clear overall enhancement relative to SCI controls (Figure 11E,F). The combined IL-4 + PPF treatment also produced robust GAP43 immunoreactivity, characterized by dense red labeling within the perilesional region and an overall appearance similar to that observed in the IL-4 group (Figure 11G,H). As expected, the uninjured spinal cord displayed comparatively low GAP43 signal relative to the IL-4-treated injured groups (Figure 11I,J).

Quantification of GAP43 immunoreactivity in the perilesional region confirmed these observations (Figure 11L). GAP43 IR was significantly increased in both the SCI + IL-4 and SCI + IL-4 + PPF groups relative to SCI, whereas the SCI + PPF group did not significantly differ from SCI. In addition, GAP43 IR in the uninjured group was lower than that observed in the SCI + IL-4 and SCI + IL-4 + PPF groups. There was no significant difference between SCI + IL-4 and SCI + PPF, as indicated in the figure, although the combined IL-4 + PPF group remained significantly elevated relative to SCI. Together, these findings indicate that IL-4-containing treatment regimens, but not PPF alone, enhance growth-associated GAP43 immunoreactivity in perilesional spinal tissue during the chronic phase after SCI, consistent with increased structural remodeling or regenerative plasticity in tissue adjacent to the lesion.

## 4. Discussion

In this rodent model of thoracic contusive SCI, systemic co-administration of PPF and IL-4 was accompanied by improved outcomes relative to vehicle treatment and, for several endpoints, relative to either monotherapy. When treatment was initiated within one hour after injury and continued for 14 days, the combined regimen was associated with improved gross and skilled locomotor recovery, reduced chronic lesion-associated inflammatory signaling, increased expression of repair-associated myeloid markers, reduced lesion cavitation, and qualitative preservation of spinal cord architecture. Collectively, these findings support the possibility that a strategy combining suppression of maladaptive inflammatory signaling with enhancement of repair-associated myeloid responses may provide greater benefit after SCI than either approach alone [13,19,20]. However, the present experiments do not establish a direct causal relationship among these outcomes and does not define the relative contribution of each component to the observed improvement, nor do they determine whether the combined effects are additive or synergistic. Such questions warrant future investigation.

One interpretation consistent with the present data is that PPF and IL-4 engage partially distinct immunoregulatory processes. IL-4 is a well-established inducer of repair-associated activation in microglia and macrophages, although its activity in the injured CNS may be constrained by a persistently inflammatory lesion environment [13,21,22,23,25]. PPF, by contrast, is a xanthine-derived glial modulator with anti-inflammatory actions, including suppression of inflammatory signaling pathways, but it is not generally considered a strong standalone inducer of reparative polarization [26,27,28,29,38,44,45,46,47]. In the present in vitro experiments, PPF increased cyclic AMP levels, reduced p-p38 MAPK expression, and enhanced IL-4-dependent ARG1 induction in TNF-α-primed BV2 microglia. These observations are consistent with, but do not prove, a model in which PPF reduces inflammatory signaling constraints and thereby permits a stronger IL-4-associated reparative response.

The in vivo data are also compatible with this interpretation. At eight weeks after SCI, lesion-associated Iba1+ myeloid cells in vehicle-treated animals showed persistent p-p38 immunoreactivity, whereas PPF-containing treatment groups showed reduced p-p38 signal. The combined treatment was additionally associated with increased ARG1 and CD206 expression relative to SCI controls. These findings are consistent with an altered lesion-associated myeloid state in the chronic injury environment. At the same time, these markers should be interpreted cautiously. Although ARG1 and CD206 are widely used as repair-associated myeloid markers, they do not by themselves define a uniformly beneficial or stable cellular phenotype. More comprehensive immune profiling will therefore be necessary to determine whether combined PPF and IL-4 treatment promotes persistent reparative programs across distinct lesion-associated myeloid populations [48,49,50].

The histological findings likewise support a measurable effect on chronic lesion pathology. Combined PPF and IL-4 treatment were associated with reduced lesion cavitation, and 3D reconstruction confirmed a significant reduction in total cavity volume at the chronic time point. Trends toward increased gray and white matter preservation were also observed, although these effects did not consistently reach statistical significance across all morphometric analyses. Accordingly, the structural data are most appropriately interpreted as evidence of reduced lesion burden and partial preservation of spinal cord architecture, rather than definitive evidence of broad tissue sparing. The association between reduced cavitation and improved locomotor recovery is notable, but the present data do not establish a direct causal relationship between these endpoints.

The degraded myelin and GAP43 findings further refine this interpretation. PPF-containing regimens reduced degraded myelin in the perilesional region, whereas IL-4 alone did not significantly alter this endpoint, suggesting that attenuation of chronic myelin pathology may be influenced primarily by the PPF component of the combined therapeutic paradigm. This interpretation is consistent with the known anti-inflammatory actions of PPF, although the present study does not distinguish between reduced ongoing myelin degradation and enhanced myelin debris clearance. In contrast, increased GAP43 immunoreactivity was more closely associated with IL-4-associated groups, particularly the combined treatment group, suggesting that the altered lesion environment may be more permissive for growth-associated plasticity. However, this observation should be interpreted conservatively, because increased GAP43 alone does not demonstrate long-distance axonal regeneration or functional circuit reconstruction.

The translational implications of this study should be interpreted with caution. Both agents were delivered systemically using a short-duration early dosing paradigm, which may be more feasible relative to more invasive approaches for SCI repair. In addition, the persistence of behavioral and histopathological benefit at 8 weeks despite cessation of treatment after 14 days suggests that time-limited modulation of the acute-to-subacute injury environment may influence chronic lesion evolution. However, the present study was conducted in a preclinical SCI model, and these findings should not be taken as direct evidence of clinical efficacy or feasibility. The study is also innovative in conceptual terms: rather than attempting to broadly suppress post-traumatic inflammation, the combined strategy is designed to reprogram the lesion immune milieu by simultaneously dampening dominant inflammatory signaling and promoting repair-associated myeloid responses.

The in vitro dosing data should likewise be interpreted within the limits of the experimental design. The concentrations of PPF used in BV2 assays were selected to interrogate signaling mechanisms relevant to cyclic nucleotide-dependent modulation of microglial phenotype, rather than to model systemic exposure achieved by the 10 mg/kg in vivo dose. Although PPF has previously been reported to cross the blood–brain barrier, pharmacokinetic measurements were not performed in the present study, and a direct quantitative relationship between in vitro concentrations and spinal cord drug exposure cannot be established. Future pharmacokinetic and pharmacodynamic studies will therefore be necessary to define the relationship between tissue drug levels, target engagement, and downstream signaling responses.

Several limitations should be acknowledged. The in vitro studies were performed in BV2 microglia and therefore provide mechanistic support but do not fully capture the complexity of the lesion-associated myeloid compartment in vivo [51]. Further, the use of cultured human microglia to replicate these findings would be important for translational relevance to clinical application based upon know species differences in microglial cell protein and miRNA production [52,53]. The present study also did not establish whether suppression of p-p38 signaling is causally required for the observed functional benefit, nor did it define the broader transcriptional or metabolic states of treatment-responsive myeloid cells. Future studies incorporating pathway-selective perturbation, single-cell and spatial profiling, and lineage-resolved analyses will be needed to more precisely define how combined PPF and IL-4 reshape lesion-associated microglial and macrophage populations over time [48,49,50]. In addition, although both male and female animals were included, the study was not designed or powered to detect sex-by-treatment interactions across all outcomes. The findings should therefore be interpreted as reflecting treatment effects in a mixed-sex cohort, while the behavioral data support the need for future studies explicitly powered to assess sex-dependent therapeutic responses.

From a therapeutic development standpoint, both components of the PPF/IL-4 strategy have prior clinical or regulated-use context that may inform future work, although neither should be viewed as de-risked for SCI translation based on the present study alone. PPF underwent prior human clinical development in dementia but was not ultimately approved for human use [54,55,56,57]. Although it is marketed for veterinary indications (including canine geriatric/age-associated conditions), providing information regarding its safety for human usage even though SCI translation would require renewed regulatory development [58]. Recombinant human IL-4 has also been administered in early-phase clinical studies, primarily in oncology, where systemic dosing feasibility and major safety liabilities were defined [59,60]. These precedents may inform future development, however, SCI-specific safety, dose optimization, pharmacokinetics, and efficacy studies remain necessary.

In summary, the present findings show that early systemic co-treatment with PPF and IL-4 was associated with improved locomotor recovery, reduced chronic lesion-associated inflammatory signaling, increased repair-associated myeloid marker expression, reduced lesion cavitation, and changes in additional tissue-level readouts after SCI. Taken together, these data support further investigation of combined PPF and IL-4 as a candidate immunomodulatory and pro-reparative strategy for SCI. More broadly, the results are consistent with the idea that therapeutic benefit after SCI may depend not only on limiting persistent inflammatory signaling but also on promoting lesion-associated immune states linked to repair.

## 5. Conclusions

Early systemic co-administration of PPF and IL-4 after contusive SCI was associated with improved locomotor recovery and with changes in the chronic lesion environment, including reduced lesion-associated inflammatory signaling and increased expression of repair-associated myeloid markers. In this model, the combined treatment was associated with reduced lesion-associated p-p38 MAPK and p65 NF-κB signaling, increased ARG1 and CD206 expression, reduced cavitation, decreased chronic myelin pathology, and findings consistent with a lesion environment that may be more permissive for growth-associated plasticity. These findings support further preclinical investigation of PPF plus IL-4 as a combinatorial immunomodulatory approach for SCI.

## Figures and Tables

**Table 1:** Primary antibodies, sources, and dilutions used for immunohistochemistry and immunoblotting.

Primary Antibody	Host	Company	Catalog Number	Dilution
Anti-Arginase 1	Rabbit	GeneTex (Irvine, CA, USA)	CTX109242	1:100
Anti-MRC1	Rabbit	Sigma-Aldrich (St. Louis, MO, USA)	HPA045134	1:100
anti-Phospho-p38 MAPK (Thr180/Tyr182) (D3F9)	Rabbit	Cell Signaling Technology (Danvers, MA, USA)	4511S	1:400
Phospho-p38 MAPK (Thr180/Tyr182)	Rabbit	Cell Signaling Technology	9211	1:1000
Anti-Iba1	Goat	Abcam (Waltham, MA, USA)	ab5076	1:500
Chicken Polyclonal to Iba1	Chicken	EnCor Biotechnology (Gainesville, FL, USA)	CPCA-Iba1	1:1000
Rabbit Polyclonal Antibody to Growth-Associated Protein 43	Rabbit	EnCor Biotechnology	RPCA-GAP43	1:1000
Chicken Polyclonal Antibody to Microtubule-Associated Protein	Chicken	EnCor Biotechnology	CPCA-MAP2	1:2000
Degraded Myelin Basic Protein	Rabbit	Millipore Sigma (St. Louis, MO, USA)	AB5864	1:1000
Myelin Basic Protein	Chicken	EnCor Biotechnology	CPCA-MBP	1:2500
Anti-β-Actin (ACTB) Antibody	Mouse	Millipore Sigma (St. Louis, MO, USA)	A1978	1:10,000

## Data Availability

The data from the current study are available from the corresponding author upon reasonable request.

## Supplementary Materials

The following supporting information can be downloaded at https://www.mdpi.com/article/10.3390/cells15070625/s1. Table S1: Post Hoc Tukey Analysis of BBB Functional Recovery Scores in Male, Female, and Combined Cohorts Following SCI.
