# Supplementary material for: Propentofylline and Interleukin-4 Modulate Lesion-Associated Myeloid Responses and Improve Functional Recovery After Spinal Cord Injury
Source: Cells. 2026 Mar 31;15(7):625. doi: 10.3390/cells15070625 (PMC13072223; doi:10.3390/cells15070625)
Supplement: Supplementary file 1 [file cells-15-00625-s001.zip › cells-4156875-supplementary.pdf]

Table S1. Post Hoc Tukey Analysis of BBB Functional Recovery Scores in Male, Female, and Combined Cohorts Following SCI

Group	Week Post-SCI	Comparison	Significance	Adjusted P Value
Male+Female BBB	1	SCI vs. SCI+IL4+PPF	***	0.0001
Male+Female BBB	1	SCI-IL4 vs. SCI+IL4+PPF	****	<0.0001
Male+Female BBB	1	SCI+PPF vs. SCI+IL4+PPF	**	0.0010
Male+Female BBB	2	SCI vs. SCI+IL4+PPF	*	0.0107
Male+Female BBB	2	SCI-IL4 vs. SCI+IL4+PPF	*	0.0251
Male+Female BBB	2	SCI+PPF vs. SCI+IL4+PPF	**	0.0052
Male+Female BBB	7	SCI vs. SCI+IL4+PPF	***	0.0004
Male+Female BBB	7	SCI-IL4 vs. SCI+IL4+PPF	*	0.0381
Male+Female BBB	7	SCI+PPF vs. SCI+IL4+PPF	*	0.0281
Male+Female BBB	8	SCI vs. SCI+IL4+PPF	****	<0.0001
Male+Female BBB	8	SCI-IL4 vs. SCI+IL4+PPF	*	0.0105
Male+Female BBB	8	SCI+PPF vs. SCI+IL4+PPF	*	0.0308
Female BBB	1	SCI vs. SCI+IL4+PPF	***	0.0001
Female BBB	1	SCI-IL4 vs. SCI+PPF	**	0.0037
Female BBB	1	SCI-IL4 vs. SCI+IL4+PPF	****	<0.0001
Female BBB	2	SCI-IL4 vs. SCI+IL4+PPF	*	0.0284
Female BBB	6	SCI vs. SCI-IL4	*	0.0456
Female BBB	7	SCI vs. SCI+PPF	*	0.0236
Female BBB	7	SCI vs. SCI+IL4+PPF	**	0.0034
Female BBB	8	SCI vs. SCI+PPF	**	0.0078
Female BBB	8	SCI vs. SCI+IL4+PPF	**	0.0029
Male BBB	1	SCI-IL4 vs. SCI+IL4+PPF	***	0.0005
Male BBB	1	SCI+PPF vs. SCI+IL4+PPF	*	0.0347
Male BBB	2	SCI-IL4 vs. SCI+IL4+PPF	*	0.0242
Male BBB	2	SCI+PPF vs. SCI+IL4+PPF	**	0.0037
Male BBB	8	SCI vs. SCI+IL4+PPF	*	0.0197
Male BBB	8	SCI-IL4 vs. SCI+IL4+PPF	*	0.0241
Male BBB	8	SCI+PPF vs. SCI+IL4+PPF	*	0.0253
